# Incomplete Urethral Duplication Associated with a Dermoid Cyst within a Vascular Hamartoma in a Female Dog

**DOI:** 10.3390/vetsci6020050

**Published:** 2019-05-30

**Authors:** Chad S. Clancy, Gordon A. Hullinger, Arnaud J. Van Wettere

**Affiliations:** Utah Veterinary Diagnostic Laboratory, School of Veterinary Medicine, Utah State University, 950 E 1400 N, Logan, UT 84341, USA; gordon.hullinger@usu.edu (G.A.H.); arnaud.vanwettere@aggiemail.usu.edu (A.J.V.W.)

**Keywords:** Urethral duplication, hamartoma, dermoid cyst

## Abstract

A seven-year-old spayed female Labrador retriever presented for necropsy following an acute history of thrombocytopenia, anemia, leukocytosis and abdominal effusion. A 2 × 3 × 10 cm, cylindrical to tubular, mottled red-to-tan mass extended from the caudal pelvic cavity caudally and ventrally under the dermis along the caudal aspect of the left pelvic limb adjacent to the semimembranosus and semitendinosus musculature. Histologic examination of the mass revealed a singular central lumen lined by urothelium that multifocally transitioned into non-keratinizing, stratified squamous epithelium associated with few hair follicles and sweat glands. The lumen was surrounded by a dense collagenous stroma containing numerous, variably sized blood vessels. The combination of lesions was consistent with a diagnosis of incomplete urethral duplication associated with a dermoid cyst and vascular hamartoma. To the authors’ knowledge, this is the first report of an incomplete urethral duplication associated with a dermoid cyst within a vascular hamartoma.

## 1. Introduction

Urethral duplication is a rare congenital anomaly infrequently diagnosed in domestic animal species. A total of five cases of urethral duplication have been reported in veterinary medicine, with four cases reported in dogs [1,2,3,4]. Urethral duplication may be complete with communication to the skin surface or may be incomplete and end blindly. In human medicine, urethral duplication is frequently reported in conjunction with one or more urogenital anomalies. A case report of a dermoid cyst within the scrotum, abutting the urethra and traversing through the corpus cavernosum has been described in a juvenile, male human [5]. A singular case report of urethral duplication associated with a dermoid cyst was described in a dog as a diverticulum lined by urothelium, which transitioned rapidly to stratified squamous epithelium associated with hair follicles [3]. To the authors’ knowledge, this report describes the first case of urethral duplication associated with a dermoid cyst within a vascular hamartoma in any species.

## 2. Case Presentation

A seven-year-old spayed female Labrador retriever presented to the Utah State Veterinary Diagnostic Laboratory for necropsy following an acute onset of thrombocytopenia and anemia coupled with a peripheral neutrophilia, lymphocytosis and monocytosis. The history indicated a previous diagnosis of coccidioidomycoses that was being treated at the time of death. Abdominal ultrasound revealed an enlarged, granular liver with irregular liver margins. The urogenital tract showed no significant ultrasonographic abnormalities. In addition to fluconazole and amphotericin B treatment for coccidioidomycoses, the patient was receiving prednisone and myocophenolate for a presumptive immune mediated anemia and thrombocytopenia, and S-adenosyl-L-methionine (SAMe) for elevated hepatic enzymes prior to presentation for necropsy. The patient died naturally at home seven days after initial presentation.

Gross necropsy revealed a hemoabdomen with approximately 1 L of serosanguineous fluid within the abdominal cavity, petechiation along the ventral abdomen and dark-red-to-purple viscous material within the entirety of the gastrointestinal tract. Originating within the pelvic fat lateral to the pelvic urethra and coursing caudally and dorsally within the subcutaneous fat overlying the left semimembranosus and semitendinosus muscles was a 2 × 3 × 10 cm, cylindrical to tubular, mottled tan-to-red mass that ended abruptly approximately one quarter to one third of the way down the pelvic limb. It was not determined at the time of necropsy if the mass directly communicated with the urinary bladder or urethra. The mass was grossly soft to spongy with a central slit-like lumen measuring up to 25 mm in diameter on cross section. The lumen continued down the entire length of the mass ending approximately 1 cm proximal to the blinded end within the pelvic limb.

**Figure 1:**
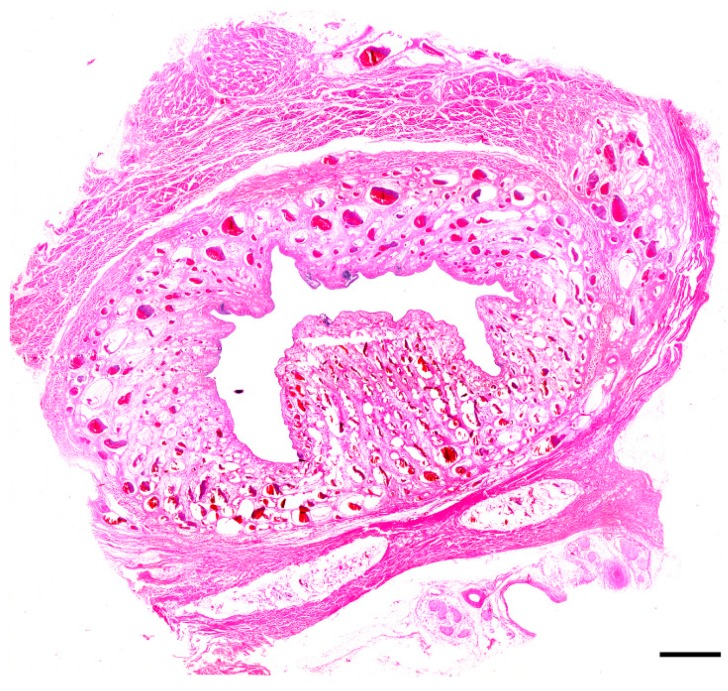
Cross section of entire mass (20x; hematoxylin and eosin). The mass is composed of a moderately dense fibrovascular stroma containing numerous blood-filled channels surrounding a lumen lined primarily by urothelium with multifocal aggregates of well-differentiated hair follicles and associated adnexal structures; bar = 2 mm.

**Figure 2:**
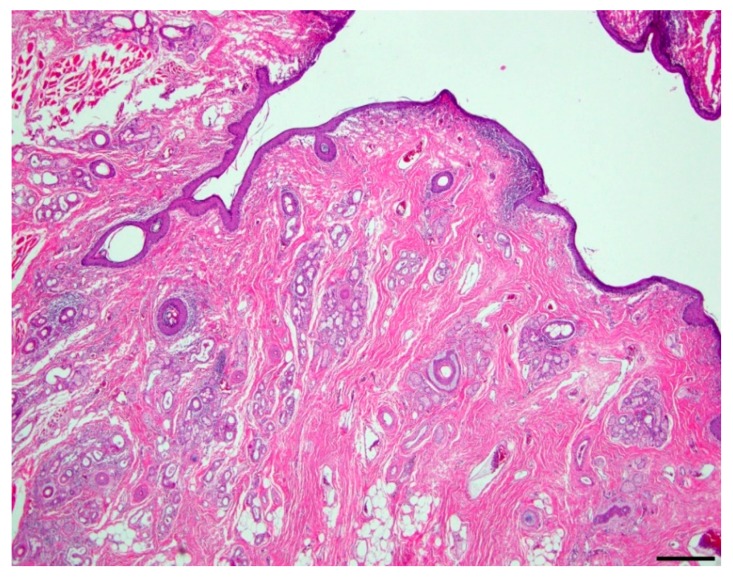
Duplicated urethra, lumen (200x; hematoxylin and eosin). Multifocally, the urothelium lining the central lumen transitions to a non-keratinizing, stratified squamous epithelium. The supporting fibrovascular stroma contains low numbers of hair follicles with associated adnexa; bar = 100 µm.

**Figure 3:**
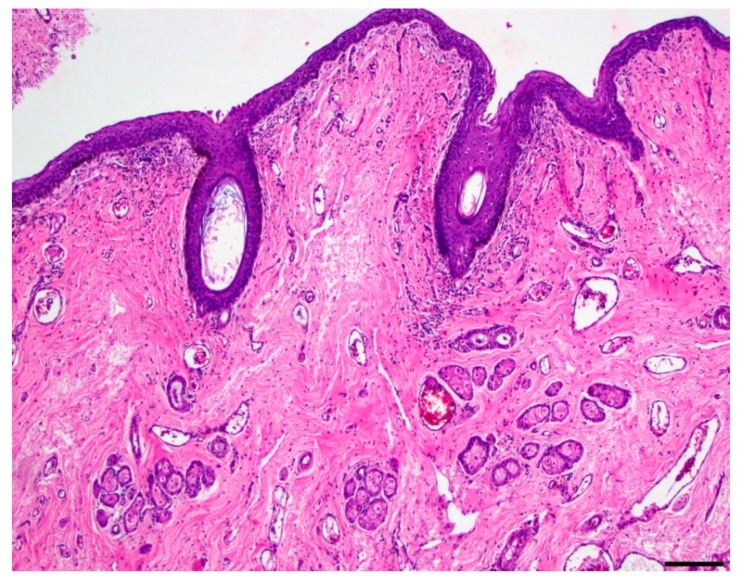
Dermoid cyst (100x, hematoxylin and eosin). The lumen is multifocally lined by non-keratinizing, stratified squamous epithelium. The supporting fibrovascular stroma contains few hair follicles, moderate numbers of sebaceous glands and fewer apocrine glands; bar = 200 µm.

**Figure 4:**
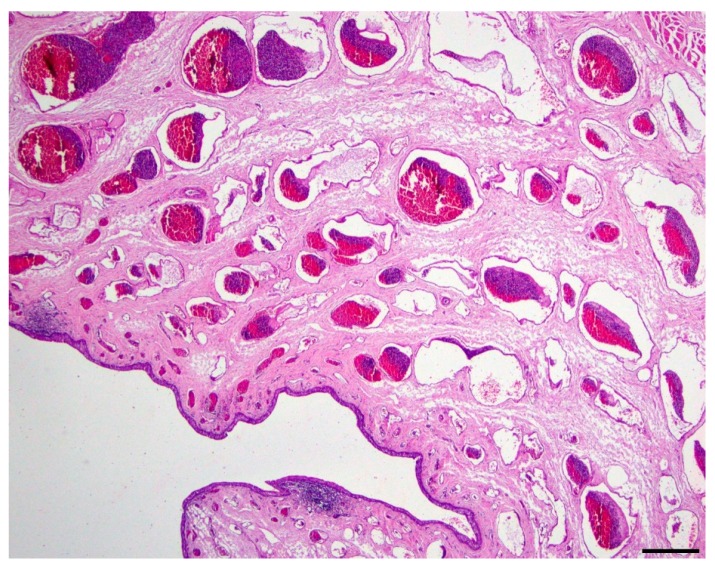
Duplicated urethra, lumen (200x, hematoxylin and eosin). The lumen of the mass is lined by urothelium, which is variably infiltrated by small-to-moderate-sized aggregates of lymphocytes and fewer plasma cells and supported by a fibrovascular stroma containing numerous, variably sized, dilated, blood-filled vascular channels; bar = 100 µm.

Microscopic evaluation of the mass revealed an unencapsulated, moderately cellular, well-demarcated expansile mass (Figure 1). The mass was centered on a lumen lined primarily by urothelium that multifocally transitioned to non-keratinizing, stratified-squamous epithelium (Figure 2). In portions of the mass lined by stratified squamous epithelium, the supporting stroma contained low numbers of hair follicles and associated sebaceous sweat glands with scattered apocrine glands (Figure 3). The lumen was surrounded by a dense collagenous stroma with few skeletal muscle bundles containing numerous, variably sized, dilated vascular channels with luminal erythrocytes (Figure 4). The vascular channels occasionally anastomosed and were lined by a single layer of endothelium. The sub-urothelial collagenous stroma was multifocally infiltrated and expanded by aggregates of lymphocytes and fewer plasma cells forming discrete aggregates (Figure 3). 

Additional gross and histologic diagnoses included cirrhosis, multiple adenohypophyseal cysts, meningioangiomatosis, splenic and bone marrow myeloid dysplasia, adrenal cortical hyperplasia and hypertrophy, lymphoplasmacytic gastritis and hemomelasma ilei. The cause of death in this dog was attributed to sequela associated with cirrhosis. Presumptive myeloid dysplasia was diagnosed on histopathologic examination of the bone marrow and splenic tissue. A complete blood count in the ante-mortem period was unavailable to confirm a concurrent diagnosis of myeloid leukemia.

## 3. Discussion

Urethral duplication is a rare lesion in veterinary medicine. This report outlines the first case of urethral duplication in a female dog. All cases of urethral duplication reported in veterinary medicine have been described in male dogs. In humans, urethral duplication is also rare, and is usually diagnosed at a young age, primarily in male patients. When diagnosed in female patients, urethral duplication is often coupled with additional urogenital abnormalities such as a prepubic sinus formation, abnormal urethral location within the vagina, duplication of the urinary bladder or renal abnormalities [6,7]. None of the previously diagnosed associated congenital urogenital abnormalities in humans were noted on gross necropsy of this female dog. 

Unlike previous reports of urethral duplication in dogs, the clinical history and medical record provided with this case did not indicate the presence of any previous urogenital abnormalities. Recurrent urinary tract infections have been previously reported with urethral duplication in a dog [3]. There were numerous lymphoid nodules lining the urothelium of the mass, suggesting chronic or recurrent antigenic stimulation, which may have been due to previous low-grade urinary tract infections from intermittent urine retention. However, it is uncertain if this duplicated urethra directly communicated with the urinary bladder in this case. Additionally, there was no evidence of current bacterial cystitis or active urinary tract inflammation in this dog. 

As with other cases of urethral duplication, multiple unrelated congenital anomalies were present in this dog. The mass ended blindly with a dermoid cyst. Dermoid cysts are rare lesions in domestic animals characterized by reduplication of haired skin including follicles and associated adnexa [8]. Dermoid cysts often occur along the midline, but may be observed in any anatomic location. The association of a dermoid cyst with a duplicated urethra has been described previously in veterinary medicine [3]. The significance of a dermoid cyst associated with a duplicated urethra is unknown in veterinary medicine. The vascular hamartoma surrounding the duplicated urethra and associated dermoid cyst was one of two vascular abnormalities. In addition to the hamartoma in the left pelvic limb, this dog also had focal meningioangiomatosis along the ventral aspect of the hypothalamus adjacent to the pituitary gland. 

The association of a vascular hamartoma in conjunction with a duplicated urethra resembles the corpus cavernosum of a penis. The external genitalia of this female dog were grossly unremarkable. Specifically, the clitoris did not appear abnormally large or prominent. However, the gonads were unavailable for histopathologic evaluation due to a previous ovariohysterectomy. A singular case report in human literature of accessory female phallic urethral diverticulum formation described similar histopathologic findings that manifested clinically as an enlarged clitoris with urinary retention [7]. The histologic description of accessory phallic diverticulum formation in a female human lacks the presence of hair follicles and associated adnexal structures. Additionally, the external genitalia of this female dog appeared physically normal at the time of necropsy, with normal urethral emptying of the bladder into the vaginal vault, suggesting that the mass observed in the caudal aspect of the pelvic limb was truly a duplication of the urethra rather than a primary disorder of sexual development. 

Urethral duplication remains a rarely diagnosed and poorly studied condition in veterinary medicine. While this condition is uncommon, urethral duplication should remain a differential diagnosis for cases of recurrent cystitis and should be evaluated in patients that present with disorders of sexual development.
